# Brap regulates liver morphology and hepatocyte turnover via modulation of the Hippo pathway

**DOI:** 10.1073/pnas.2201859119

**Published:** 2022-04-27

**Authors:** Christina Priest, Rohith T. Nagari, Lara Bideyan, Stephen D. Lee, Alexander Nguyen, Xu Xiao, Peter Tontonoz

**Affiliations:** ^a^Department of Pathology and Laboratory Medicine, David Geffen School of Medicine, University of California, Los Angeles, CA 90095;; ^b^Department of Biological Chemistry, David Geffen School of Medicine, University of California, Los Angeles, CA 90095;; ^c^Vatche and Tamar Manoukian Division of Digestive Diseases, Department of Medicine, David Geffen School of Medicine, University of California, Los Angeles, CA 90095

**Keywords:** liver, ubiquitin ligase, Hippo pathway

## Abstract

Regulation of hepatocyte proliferation and liver morphology is of critical importance to tissue and whole-body homeostasis. However, the molecular mechanisms that underlie this complex process are incompletely understood. Here, we describe a role for the ubiquitin ligase BRCA1-associated protein (BRAP) in regulation of hepatocyte morphology and turnover via regulation of MST2, a protein kinase in the Hippo pathway. The Hippo pathway has been implicated in the control of liver morphology, inflammation, and fibrosis. We demonstrate here that liver-specific ablation of *Brap* in mice results in gross and cellular morphological alterations of the liver. *Brap*-deficient livers exhibit increased hepatocyte proliferation, cell death, and inflammation. We show that loss of BRAP protein alters Hippo pathway signaling, causing a reduction in phosphorylation of YAP and increased expression of YAP target genes, including those regulating cell growth and interactions with the extracellular environment. Finally, increased Hippo signaling in *Brap* knockout mice alters the pattern of liver lipid accumulation in dietary models of obesity. These studies identify a role for BRAP as a modulator of the hepatic Hippo pathway with relevance to human liver disease.

## Significance

The liver has a complex cellular architecture that is essential for its function. In this work, we show that BRAP, a ubiquitin ligase of poorly understood function, is required for maintenance of proper liver morphology. BRAP deletion in the liver causes disruption of its normal architecture and inflammation due to altered expression of genes involved in cell growth and extracellular interactions. This work sheds light on the mechanisms that maintain proper liver structure and has implications for liver disease.

Coordination of cellular metabolism and growth pathways is critical for proper tissue homeostasis in the liver. Dysregulation of these processes contributes to lipid excess, impaired regeneration, and tissue inflammation. Understanding the mechanisms that mediate this coordination has clinical implications for the development of nonalcoholic fatty liver disease, cirrhosis, and hepatocellular carcinoma. One class of proteins that may serve this function is ubiquitin ligases. Members of this family are known to modulate fatty acid and cholesterol homeostasis in the liver (1, 2). BRCA1-associated protein (BRAP), a poorly characterized E3 ligase, is expressed in the liver, but its role in hepatic physiology is unknown.

BRAP was originally identified in a yeast two-hybrid screen for proteins interacting with the BRCA1 tumor suppressor (3). However, BRAP has not been shown to act as a tumor suppressor, and its physiological role is incompletely understood. BRAP has autoubiquitination activity, suggesting that it is a functional ubiquitin ligase (4), but its targets are not well defined. Previous work has suggested that BRAP functions in the central nervous system to control development by modulating the MAP kinase pathway, partially through increasing ubiquitination of SKP2 (5, 6). However, BRAP is expressed at comparable levels in the liver and has been associated with low-density lipoprotein and myocardial infarction risk in several genome-wide association studies, implying that it has additional, uncharacterized, tissue-specific functions (7, 8).

Here, we describe a role for BRAP in regulation of hepatocyte morphology and turnover via regulation of MST2, a protein kinase in the Hippo pathway. Loss of BRAP results in depletion of MST2, which allows the transcription factor YAP to translocate to the nucleus and promote transcription of genes involved in cell growth and cytoskeletal remodeling. We demonstrate that liver-specific ablation of *Brap* in mice results in gross and cellular morphological alterations of the liver. *Brap* knockout livers exhibit increased hepatocyte proliferation, cell death, and inflammation. Additionally, mice lacking *Brap* expression in hepatocytes (*Brap LKO*) fed Western or nonalcoholic steatohepatitis–inducing (NASH) diets exhibit altered lipid accumulation. These results establish BRAP as a regulator of hepatic morphology both at baseline and during response to dietary stress.

## Results

### Hepatic Brap Expression Is Important for Liver Morphology.

**Fig. 1.**
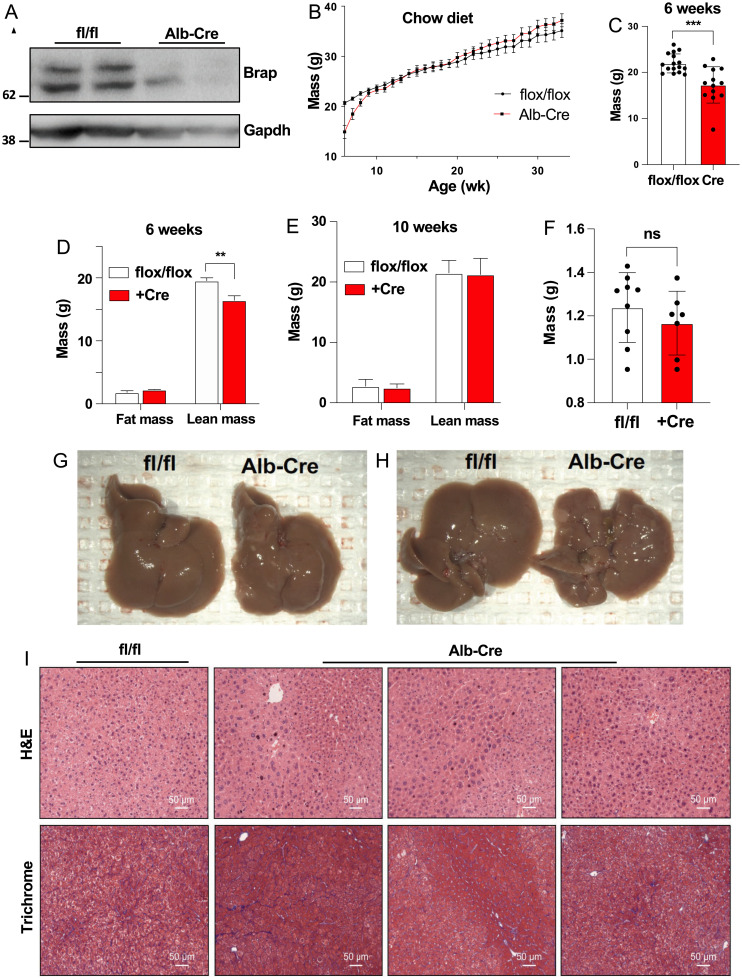
Characterization of *Brap LKO* mice. (*A*) Anti-Brap and anti-GAPDH Western blots of *Brap LKO* and WT mice. (*B*) Body weights of male *Brap LKO* mice and WT littermate flox/flox controls, from 6 wk to 35 wk, fed a chow diet. (*C*) Body weights of *Brap LKO* and WT mice at 6 wk. (*D*) Fat and lean mass of *Brap LKO* and WT mice at 6 wk. (*E*) Fat and lean mass of *Brap LKO* and WT mice at 10 wk. (*F*) Liver weights of *Brap LKO* and WT mice at 18 wk. (*G* and *H*) Gross appearance of male *Brap LKO* livers (*G*) and WT controls (*H*) at 18 wk. (*I*) Hematoxylin and eosin (H&E) (*Top*) and trichrome (*Bottom*) staining of male *Brap LKO* and WT control livers at 18 wk. All images are representative of three replicate mice. Alb, albumin; *P < 0.005; ***P < 0.0005; ns, not significant.

Prior work determined that whole-body knockout of *Brap* is lethal at embryonic day 12 (6). We chose to create a tissue-specific knockout model in order to study the function of this gene in the liver. We developed *Brap*-floxed (*Brap*^F/F^) mice in collaboration with the University of California Irvine Transgenic Mouse Facility (*SI Appendix*, Fig. S1) and crossed *Albumin*-Cre transgenic mice with *Brap^F/F^* mice to generate *Brap LKO* mice. Western blot with an anti-BRAP antibody indicated the loss of two bands of similar molecular weight at ∼65 kDa (predicted, 67 kDa) in the knockout mice (Fig. 1*A*). The *Brap LKO* mice had reduced body weight at 6 wk of age, but by age 10 wk had a body weight and composition equivalent to that of *Brap^F/F^* control mice (Fig. 1 *B* and *C*). This initial reduction in body mass was attributable to a reduction in lean body mass as measured by MRI (Fig. 1 *D* and *E*). A similar, albeit milder, body weight phenotype was observed in female mice (*SI Appendix*, Fig. S2).

Livers from 18-wk-old *Brap LKO* mice had similar mass as control livers (Fig. 1*F*) but exhibited gross morphological changes (Fig. 1 *G* and *H*). On gross examination, *Brap LKO* livers had a bumpy appearance, with abundant light-colored nodules. Staining with hematoxylin and eosin and Masson’s trichrome further revealed altered liver structure in *Brap LKO* mice compared with floxed littermate controls (Fig. 1*I*). *Brap LKO* livers contained interspersed nodules of hepatocytes of heterogeneous size and shape, as well as increased fibrosis. These findings suggest that BRAP is required for maintenance of normal liver architecture.

### Increased Hepatocyte Proliferation, Apoptosis, and Inflammation in Brap-Deficient Mice.

**Fig. 2.**
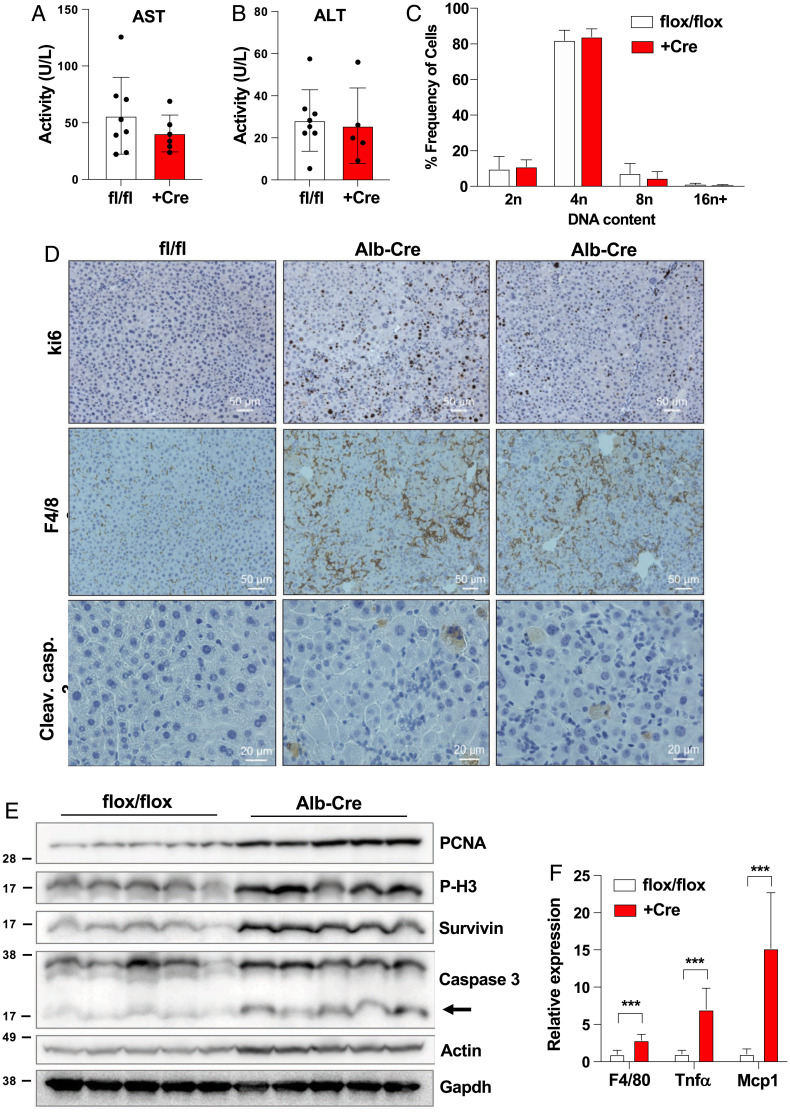
Analysis of *Brap LKO* mouse livers. (*A* and B) Serum aspartate transaminase (AST) and alanine transaminase (ALT) levels of *Brap LKO* mice and WT control mice. (*C*) Ploidy of primary hepatocytes from *Brap LKO* and WT mice. (*D*) *K*_i_-67, cleaved caspase (Cleav. casp.) 3, and F4/80 staining of *Brap LKO* and WT mice livers. (*E*) Western blot for PCNA, P-H3, survivin, cleaved caspase 3, and actin in *Brap LKO* and WT mice. (*F*) Relative expression via qPCR of F4/80, TNF-α, and Mcp1 in *Brap LKO* and WT mice. ***P < 0.0005.

*Brap LKO* mice did not show elevation in plasma alanine transaminase or aspartate transaminase at 6 wk of age, suggesting the absence of substantial ongoing injury (Fig. 2 *A* and *B*). Additionally, despite the alterations in cell and nuclear size visible in liver sections (Fig. 1*G*), we observed no difference in hepatocyte ploidy as measured by propidium iodide staining by fluorescence-activated cell sorting (Fig. 2*B* and *SI Appendix*, Fig. S3). In order to determine the cause of the altered morphology in *Brap LKO* livers, we assayed for markers of proliferation, apoptosis, and inflammation. We observed increased staining of KI-67 (a marker of proliferation), cleaved caspase 3 (a marker of apoptosis), and F4/80 (a macrophage marker) in the *Brap LKO* livers (Fig. 2*D*). These data suggest increased hepatocyte turnover accompanied by inflammation. These results were confirmed by Western blot analysis of liver lysates from 6-wk-old mice, which had marked increases in proliferative markers (PCNA and phospho-H3) as well as apoptotic markers (cleaved caspase-3 and survivin) in *Brap LKO* livers (Fig. 2*E*). Gene expression analysis by qPCR also showed an increased F4/80, TNF-α, and Mcp-1 expression in *Brap LKO*s, suggesting an increase in infiltration of immune cells and inflammation (Fig. 2*F*).

### Loss of Brap Alters Expression of Genes Regulating Interaction with the Extracellular Environment.

**Fig. 3.**
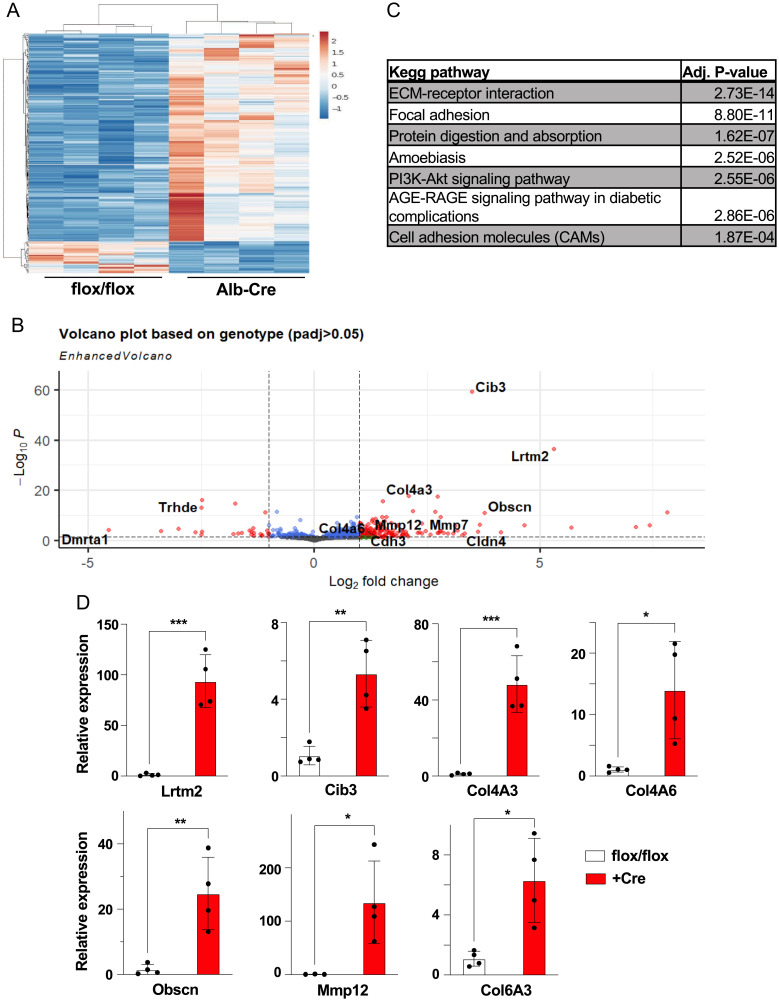
RNA sequencing (RNAseq) of livers from *Brap LKO* and WT mice. *(A*) Heat map of 452 differentially up-regulated and 170 significantly differentially down-regulated genes in *Brap LKO* livers relative to WT livers. (*B*) Volcano plot of statistical significance against log2 fold-change of gene expression between WT and *Brap LKO* livers. (*C*) KEGG analysis of most significantly up-regulated pathways in *Brap LKO* livers. (*D*) qPCR validation of top RNAseq hits (*n* = 3 to 4). *P < 0.05; **P < 0.01; ***P < 0.0001. Alb, albumin; padj, adjusted *P* value.

To identify the gene expression changes underlying the hepatic phenotypes of *Brap LKO* mice, we performed bulk RNA sequencing. In total, 452 genes were significantly up-regulated and 170 were significantly down-regulated in the livers of *Brap LKO* mice (Fig. 3*A*). Further analysis identified pathways that were differentially expressed. The Kyoto Encyclopedia of Genes and Genomes (KEGG) pathways most differentially expressed between *Albumin*-Cre mice and floxed control mice were predominantly related to the cytoskeleton and extracellular matrix (ECM), including ECM–receptor interaction and focal adhesion (Fig. 3*C*). Supporting this, among the most differentially expressed individual genes were collagens *Col4a3*, *Col4a6*, and *Col6a3*, as well as ECM-related genes such as *Obscn* and *Mmp12* (Fig. 3*B*). We quantified the fold change of these genes with qPCR (Fig. 3*D*). These results suggest that the mechanisms underlying morphological alterations in *Brap LKO* livers likely include altered interactions with the extracellular environment.

### Loss of Brap Alters Hippo Signaling and YAP Target Gene Expression.

**Fig. 4.**
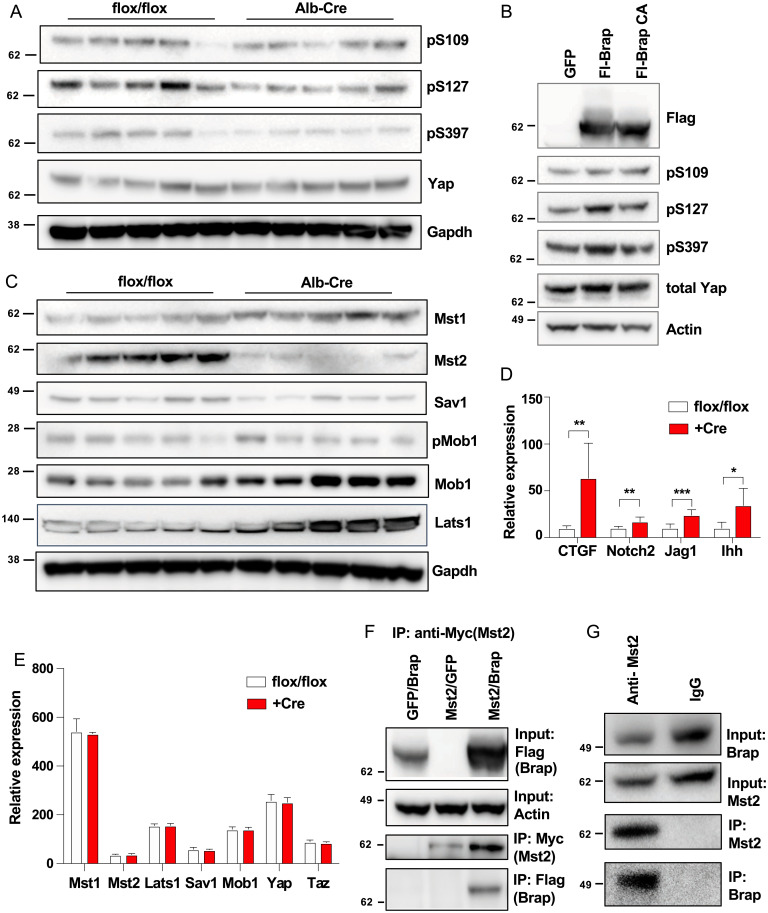
Alterations in Hippo pathway signaling in *Brap LKO* mice and characterization of Brap/Mst2 interaction. (*A*) Yap phosphorylation in livers of WT and *Brap LKO* mice. (*B*) Yap phosphorylation in HepG2 cells overexpressing Brap WT or an E3 ligase–deficient mutant BRAP (BrapCA). (*C*) Western blot of Hippo pathway proteins in WT and *Brap LKO* mice. (*D*) Relative expression via qPCR of Yap target genes in livers of WT and *Brap LKO* mice. (*E*) mRNA expression (from RNA-sequencing counts) of Hippo pathway genes. (*F*) Immunoprecipitation (IP) with anti-Mst2 antibody or IgG control of HEK293T cell lysates. (*G*) Immunoprecipitation with anti-Myc antibody of lysates from HEK239T cells transfected with Myc-Mst2 and either GFP or Flag-Brap. Alb, albumin. *P < 0.05; **P < 0.05; ***P < 0.0005.

The changes in expression of cytoskeletal and ECM proteins seen in *Brap LKO* mice led us to hypothesize that BRAP might regulate cell–cell or cell–ECM interactions. The Hippo pathway is an established regulator of liver size and structure that responds to physical cues transmitted by cell–cell and cell–ECM contacts. Phosphorylation of YAP, one of the main transcriptional effectors of this pathway, prevents its entry to the nucleus and reduces expression of genes such as connective tissue growth factor (CTGF); cysteine-rich angiogenic inducer 61; notch 2; jagged 1; and Indian hedgehog (9–12). To determine if Hippo pathway signaling was with altered *Brap* knockout, we interrogated the protein levels and posttranslational modifications of key players in this pathway. *Brap LKO* liver displayed normal levels of total YAP protein but decreased phosphorylation of YAP S109, S127, and S397, suggesting increased nuclear localization. Furthermore, YAP phosphorylation was accompanied by increased expression of the aforementioned YAP target genes (Fig. 4*D*).

In order to determine if the observed increase in YAP transcriptional activity was a cell-autonomous effect, we sought to determine if alteration of BRAP levels in cultured cells induced changes in YAP translocation. Indeed, overexpression of wild-type (WT) BRAP in HepG2 cells was sufficient to increase YAP phosphorylation, while overexpression of an E3 ligase–deficient mutant BRAP was not (Fig. 4*B*) (9).

We next sought to identify specific alterations in Hippo pathway signaling that could be responsible for alteration of Yap phosphorylation in the absence of Brap. Upstream of YAP, MST1 and MST2 kinases phosphorylate SAV1 and MOB1, which then recruit LATS1 and LATS2 kinases to phosphorylate YAP (13). We analyzed levels of these proteins by Western blot and found that MST1 and total MOB1 levels were elevated in *Brap LKO* mice. At the same time, levels of phospho-MOB1, SAV1, LATS1, and, most dramatically, MST2 were decreased (Fig. 4*C*). Messenger RNA (mRNA) levels of MST1/2, LATS1, MOB1, YAP, and TAZ proteins were not different between groups, suggesting that BRAP regulation of the Hippo pathway is posttranscriptional (Fig. 4*E*).

The dramatic difference in MST2 levels between WT and *Brap LKO* mice led us to hypothesize that BRAP may interact with MST2 directly. When Myc-MST2 and Flag-BRAP were coexpressed in HEK293T cells, pull down of MST2 using the Myc epitope confirmed an interaction with Flag-BRAP (Fig. 4*F*). Furthermore, endogenous MST2 from HEK239T coimmunoprecipitated with endogenous BRAP, verifying that the two proteins are found in a complex intracellularly (Fig. 4*G*). Taken together, these data support the conclusion that BRAP’s regulation of liver structure and morphology occurs via its modulation of the Hippo pathway, specifically through its interaction with MST2. Brap deletion leads to a decrease in MST2 protein levels, which prevents YAP phosphorylation and ultimately results in increased expression of proliferative and profibrotic YAP target genes and a grossly nodular and hyperplastic liver.

### Loss of Brap Affects Hepatic Responses to NASH and Western Diets.

**Fig. 5.**
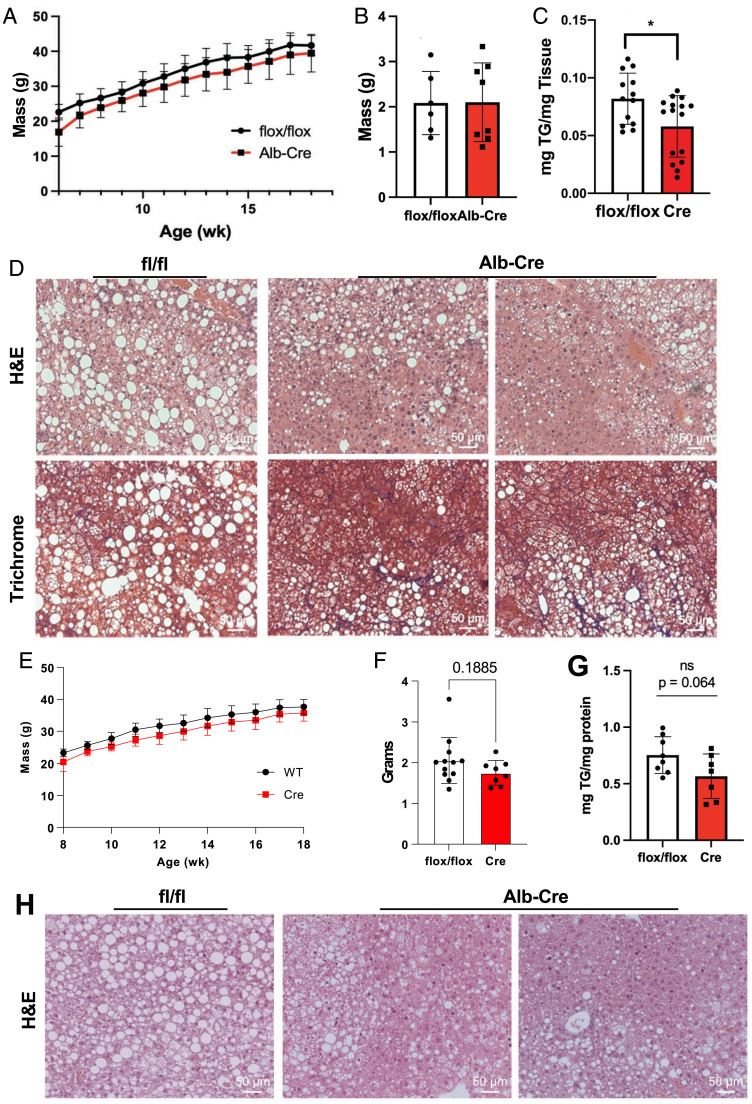
*Brap LKO* mice exhibit alterations in triglyceride accumulation upon pathological diet feeding. (*A*) Weights of *Brap LKO* and WT mice on a 14-wk high-fat, high-sucrose NASH diet. (*B* and *C*) Liver weights (*B*) and triglyceride content (*C*) of *Brap LKO* and WT mice after 16 wk of NASH-diet feeding. (*D*) H&E and trichrome staining of livers of *Brap LKO* and WT mice after 16 wk of NASH-diet feeding. (*E*) Weights of *Brap LKO* and WT mice fed a 10-wk Western diet. (*F* and *G*) Liver weight (*F*) and triglyceride (*G*) content of *Brap LKO* and WT mice after a 10-wk Western diet. (*H*) H&E-stained livers of *Brap LKO* and WT mice after 10 wk of Western diet feeding. Alb, albumin; ns, not significant; TG, triglyceride. *P < 0.05.

The baseline liver morphology and gene expression we observed in *Brap LKO* mice fed a chow diet led us to explore how these mice would respond to the stress of pathological diets. Specifically, we sought to understand how Brap-deficient livers responded to diets that promote steatosis and fibrosis. To this end, we challenged *Brap LKO* mice and *Brap^F/F^* littermate control mice with a Western diet and a NASH-inducing high-fat, high-sucrose diet with added cholesterol. Plasma triglyceride levels were not different between groups (*SI Appendix*, Fig. S4*A*). *Brap LKO* mice fed a NASH-inducing diet for 16 wk had similarly sized livers but accumulated less hepatic triglycerides than did their floxed control counterparts (Fig. 5 *A*–*C*). Sectioning and staining revealed that the pattern of lipid accumulation was also markedly different between groups. While control livers accumulated large lipid droplets that were distributed uniformly, *Brap LKO* livers had discrete regions of lipid accumulation alternating with regions of small, hyperplastic hepatocytes (Fig. 5*D*). Furthermore, trichrome staining revealed enhanced fibrosis in livers lacking Brap (Fig. 5*D*).

*Brap LKO* mice fed a Western diet for 10 wk displayed a phenotype similar to those fed a NASH diet. Plasma triglyceride levels were not different between groups (*SI Appendix*, Fig. S4*A*), but *Brap LKO* livers from mice fed a Western diet weighed slightly less than that of control mice, and there was a trend toward less triglyceride accumulation (Fig. 5 *E*–*G*). Histologically, regions with lipid accumulation in livers of *Brap LKO* mice fed a Western diet were disrupted by nodules of small hepatocytes, similar to the findings in livers of mice fed the NASH diet (Fig. 5*H*). Together, these data demonstrate that BRAP not only regulates liver morphology at baseline but also affects liver remodeling in response to pathological stress.

## Discussion

The Hippo pathway integrates microscopic, hepatocyte cytoskeletal architecture with macroscopic traits such as whole-liver size and morphology. Hippo signaling has been implicated in control of an array of processes within the liver, ranging from regeneration to NASH to cancer (9, 14–19). Here, we have identified BRAP as a regulatory node within this pathway through its regulation of the kinase MST2. Liver-specific Brap deletion in mice leads to a grossly nodular liver. Microscopically, *Brap LKO* livers are inflamed, apoptotic, and hyperplastic. This phenotype results, at least in part, from increased Hippo pathway signaling that drives expression of cytoskeletal and ECM-remodeling proteins such as CTGF, Indian hedgehog, notch 2, and jagged 1. In WT mice, BRAP interacts with MST2 kinase and maintains YAP phosphorylation, thereby restraining Hippo pathway activity. Loss of BRAP leads to the depletion of MST2, increased YAP transcriptional activity, and expression of profibrotic and hepatocyte proliferative genes.

In addition to affecting gross morphology of the liver, loss of *Brap* led to increased hepatocyte proliferation and apoptosis, suggesting an increase in hepatocyte turnover. There was also immune cell infiltration and increased inflammatory marker expression, suggesting an inflammatory response to *Brap* deletion. Based on gene expression analysis of the liver, it seems likely that the increased turnover and inflammation seen in *Brap LKO* mouse livers are secondary to Yap-mediated transcription of cytoskeleton-remodeling genes. However, it is possible that loss of BRAP causes cell death, which contributes to the inflammation and fibrosis seen in *Brap LKO* mice. Future work will be needed to dissect this. Regardless, the increase in fibrotic and inflammatory signaling in *Brap LKO* livers provides insight into the role of BRAP in regulation of normal liver homeostasis. This role also appears to also be important in the response to dietary stress, as revealed by Western and NASH diet challenges.

Several questions remain regarding the role of BRAP in Hippo signaling. Foremost, the mechanism behind MST2 protein loss in *Brap LKO* mice requires further investigation. Prior work has indicated that BRAP is a functional ubiquitin ligase; however, the role ubiquitination in MST2 regulation remains unclear. While BRAP’s ubiquitin ligase activity is required for alteration of YAP transcriptional activity in HepG2 cells, loss of BRAP results in reduction of MST2 protein levels, contrary to what would be expected if BRAP was regulating MST2 itself via proteasomal degradation. It is possible that increased MST2 degradation and/or loss of MST2 sequestration results from the altered ubiquitination of another yet-unidentified protein. Accordingly, more work into the exact nature of the BRAP-MST2 interaction, and whether it is direct or requires other factors, could help illuminate the underlying regulatory mechanisms.

Hepatocyte glycogen droplets have recently been proposed to act as Hippo pathway signaling hubs (20). Intriguingly, we have observed that suppression of BRAP with short hairpin RNA decreases liver glycogen levels (*SI Appendix*, Fig. S4*B*). These findings raise the exciting possibility that glycogen could play a role in mediating the BRAP-MST2 interaction. CRISPR screening has identified *Brap* as one of a select few sex-specific essential genes in developing mouse livers; our own observations indicate that male *Brap LKO* mice display more dramatic changes in liver structure than do female mice (21). Thus, the sex-specific roles of *Brap* also invite future inquiry.

Finally, modulation of *Brap* may be a possible avenue to modulate Hippo pathway response under these conditions, which has relevance to human liver disease, including NASH and nonalcoholic fatty liver disease. Our observation that YAP transcriptional activity is responsive to BRAP in human HepG2 cells indicates that control of the Hippo pathway by BRAP is conserved in humans.

## Materials and Methods

### Experimental Models.

The Brap^F/F^ mice were generated using Crispr/Cas9 to insert flox sites flanking exons 2 and 3 of the *Brap* gene. These mice were generated at the UC Irvine Transgenic Mouse Facility on a C57BL/6J background. The mouse studies conducted at University of California Los Angeles (UCLA) were reviewed and approved by the Chancellor's Animal Research Committee. All mice were housed in specific pathogen-free, climate-controlled facilities maintained at 22 °C on 12-h light/dark cycles. *Albumin*-Cre transgenic mice were obtained from Jackson Laboratory.

### Mouse Studies.

All studies were carried out using male mice maintained in a climate-controlled vivarium on a 12-h light/dark cycle at a temperature of 22 °C (22). Unless otherwise noted, animals were group-housed mice. Both sexes of mice were used for pilot studies; however, female mice exhibited milder phenotypes and were resistant to diet-induced obesity. For conditional knockout studies with Western diet–fed mice, 5- to 6-wk-old mice were challenged with diet for 10 wk. Body mass was determined weekly. For studies with the NASH diet, 5- to 6-wk-old mice were fed the NASH diet for 16 wk. Body mass was determined weekly. Adiposity was determined by MRI (EchoMRI 3-in-1) at baseline and prior to termination. At the conclusion of each study, mice were killed by isoflurane overdose and exsanguinated by cardiac puncture before cervical dislocation. Blood was collected in ethylenediaminetetraacetic acid–coated tubes, and plasma was separated by centrifugation at 2,000*g* for 15 min. Tissues were snap frozen in liquid nitrogen or fixed in 4% paraformaldehyde. For adenoviral overexpression or knockdown of Brap, age-matched (8- to 10-wk-old) male mice were injected with 1.5 × 10^9^ PFU of mouse Brap (mBrap) or shRNA against mouse Brap (shBrap)-expressing adenovirus by tail-vein injection. Mice were killed 7 to 10 d after injection and tissue was collected as described previously in this section.

### Generation and Amplification of Adenoviral Particles.

For adenoviral vector production of mBrap, the sequence encoding *Brap* was amplified from complementary DNA (cDNA) reversely transcribed from murine total mRNA and cloned into pENTR1A. We generated pAd/CMV/V5-DEST-mBrap by recombination of pENTR1A-mBrap and pAd/CMV/V5-DEST using the Gateway system (Life Technologies).

For viral knockdown of Brap, pAD/BLOCK-iTDEST-shBrap was generated using the pAdBLOCK-iT kit (Life Technologies). Briefly, several oligonucleotides targeting *Brap* were designed with BLOCK-iT RNAi designer software (https://rnaidesigner.thermofisher.com/rnaiexpress/; Life Technologies) and cloned into pU6- ENTR. The resulting pU6-ENTR-shBrap plasmids were tested for their ability to inhibit *Brap* expression in transient transfection experiments in Hepa 1-6 cells (American Type Culture Collection). The construct showing the greatest inhibition was recombined with pAD/BLOCK-iTDEST, using the Gateway system (Life Technologies). Adenovirus particles were generated by transfection of pAd/CMV/V5-DEST-mBrap and pAD/BLOCK-iTDEST-shBrap into 293A cells (Life Technologies) using Fugene 6 transfection reagent (Promega) according to manufacturers’ instructions. Viruses were amplified, purified, and titered by Viraquest.

### Gene Expression.

RNA was isolated from frozen tissues with TRIzol (Life Technologies) according to manufacturer’s instructions. Gene expression was determined by real-time RT-qPCR (Bio-Rad) using an Applied Biosystems Quant Studio 6 Flex. Results were normalized to the average expression levels of 36B4.

### Western Blot Analysis.

For protein isolation, tissues were processed with a Dounce homogenizer using radioimmunoprecipitation assay buffer buffer (Boston BioProducts) that contained both phosphatase and protease inhibitors (Roche) (22). Bis-Tris gels were used to separate proteins by electrophoresis prior to transfer to polyvinylidene difluoride membranes for blotting. To reduce nonspecific binding, membranes were blocked in a solution of 5% nonfat milk in phosphate-buffered saline (PBS) prior to incubation with specific antibodies. Secondary antibodies used were horseradish peroxidase–conjugated anti–mouse and anti–rabbit IgG (Jackson Laboratory). Signals were visualized using Immobilon Forte Western HRP Substrate (EMD Millipore).

### Immunoprecipitation.

Cells were lysed in immunoprecipitation buffer (20 mM Tris, 137 mM NaCl, 1% Triton ×100, pH 7.4) supplemented with phenylmethylsulfonyl fluoride and protease and phosphatase inhibitors. The concentration of proteins was determined using Pierce 660nm Protein Assay Reagent (Thermo Scientific) according to manufacturer's instructions. Protein (1 μg) was incubated with primary antibody for 2 h with end-over rotation at 4 °C. Protein A/G Sepharose beads (Santa Cruz Biotechnology) were washed twice with lysis buffer and added to lysates for an additional 4 h at 4 °C. Beads were washed three times and proteins were eluted by mixing an equal volume of 2× sample buffer and boiling at 95 °C for 5 min.

### Histology.

Tissues were fixed in 4% paraformaldehyde for 72 h and stored in 70% ethanol before being mounted in paraffin. The 10-µm sections were cut and stained with hematoxylin and eosin or Masson’s trichrome by the UCLA Translational Pathology Core. Immunohistochemistry was performed by the UCLA Translational Pathology Core.

### FACS Analysis.

Primary hepatocytes were isolated from 6- to 8-wk-old mice as previously described (20). Cells in suspension were fixed in 70% ethanol before staining with propidium iodide staining solution (BD Biosciences) per manufacturer’s instructions. They were analyzed using a BD FACSVerse machine and FlowJo software (BD Biosciences).

### Triglyceride Extractions.

Liver triglycerides were extracted as previously described (2) and measured by Wako L-Type TG M kit.

### Plasmids and Transfection.

For transient transfections and viral vector production of the *BRAP*, the sequence encoding Brap was amplified from cDNA reversely transcribed from human total mRNA. The resulting sequence fragment was tagged with a FLAG polypeptide sequence (DYKDDDDK) at the N terminus and then cloned into a pDONR221 entry vector using the Gateway system (Life Technologies). The C264A mutation of *BRAP* was introduced using the Q5 site-directed mutagenesis kit (New England BioLabs). The sequences in the entry clones were then transferred by LR recombination into pcDNA-DEST47 using the Gateway system (Life Technologies) for transient transfections. HA-MST1(plasmid no. 12203) and Myc-MST2 (plasmid no. 12205) plasmids were gifts from Jonathan Chernoff , Cancer Biology Program, Fox Chase Cancer Center, Philadelphia, PA (Addgene).

HEK293T cells were obtained from the American Type Culture Collection. They were previously verified by short tandem repeat testing and were confirmed to be mycoplasma-free by regular testing. Cells were cultured in Dulbecco’s Modified Eagle medium supplemented with 10% fetal bovine serum (Invitrogen), 100 U/mL penicillin, and 100 U/mL streptomycin. Cells were incubated at 37 °C in a humidified incubator containing 5% CO_2_. Transfections were performed using Fugene 6 transfection reagent (Promega) according to manufacturer’s instructions.

### RNA Sequencing and Data Analysis.

RNA was isolated from frozen tissues with TRIzol (Life Technologies) according to manufacturer’s instructions, followed by column purification (RNeasy Kit; Qiagen). RNA was quantified using Qubit Fluorometric Quantitation (Thermo Fisher). Total RNA libraries were made with the NuGEN Universal Plus mRNA kit. Libraries were sequenced single end (50 bp) on a HiSeq3000 instrument.

Data quality analysis was performed via FastQC (23). The reads were aligned to the mm10 genome using STAR [version 2.6.0c (24)]. Alignments were visualized using samtools (25) and the IGV browser (26). Differential expression analysis was performed with DESeq2 (27), and genes were classified as significantly regulated if the adjusted *P* value was <0.05. Genes were annotated using *biomaRt* package in R (https://www.R-project.org/) (28, 29). Plots and heat maps were created in R using *EnhancedVolcano* and the *ClustVis* web tool (30). Pathway analysis was performed with KEGG (31).

### Glycogen Assay.

To assess liver glycogen levels, ∼50 mg of previously snap-frozen liver was weighed and homogenized in 500 µL of ice-cold PBS. Homogenates were heated to 95 °C and cooled on ice before centrifugation for 10 min at room temperature to remove precipitate. Supernatant was assayed for glycogen content using the Glycogen Assay Kit (Sigma-Aldrich) according to manufacturer’s instructions. Glycogen content was normalized to initial liver weight.

## Supplementary Material

Supplementary File

## Data Availability

RNA sequencing data from *Brap* LKO mouse livers are available in the Gene Expression Omnibus (accession no. GSE196012) (32).
