# Supplementary material for: Brap regulates liver morphology and hepatocyte turnover via modulation of the Hippo pathway
Source: Proc Natl Acad Sci U S A. 2022 Apr 27;119(18):e2201859119. doi: 10.1073/pnas.2201859119 (PMC9171358; doi:10.1073/pnas.2201859119)
Supplement: Supplementary File [file pnas.2201859119.sapp.pdf]

Supplemental Figure Legends:

Figure S1: Targeting strategy for generation of Brap LKO mice.

*Figure S2: (*A*)* Body weights of female Brap LKO and WT mice. Body weights of female Brap LKO and WT mice at 6 weeks. Fat and lean mass of female Brap LKO and WT mice (determined by MRI) at 6 weeks *(*C*)* and 10 weeks *(*D*). (*E*)* Liver weights of female Brap LKO mice at 10 weeks.

Figure S3: Gating strategy for propidium iodide study of primary hepatocytes isolated from Brap LKO and WT mice

Figure S4: (A) Plasma triglyceride levels in fed western or NASH diet. (B) shRNA-mediated knockdown of Brap in mouse liver alters liver glycogen levels.

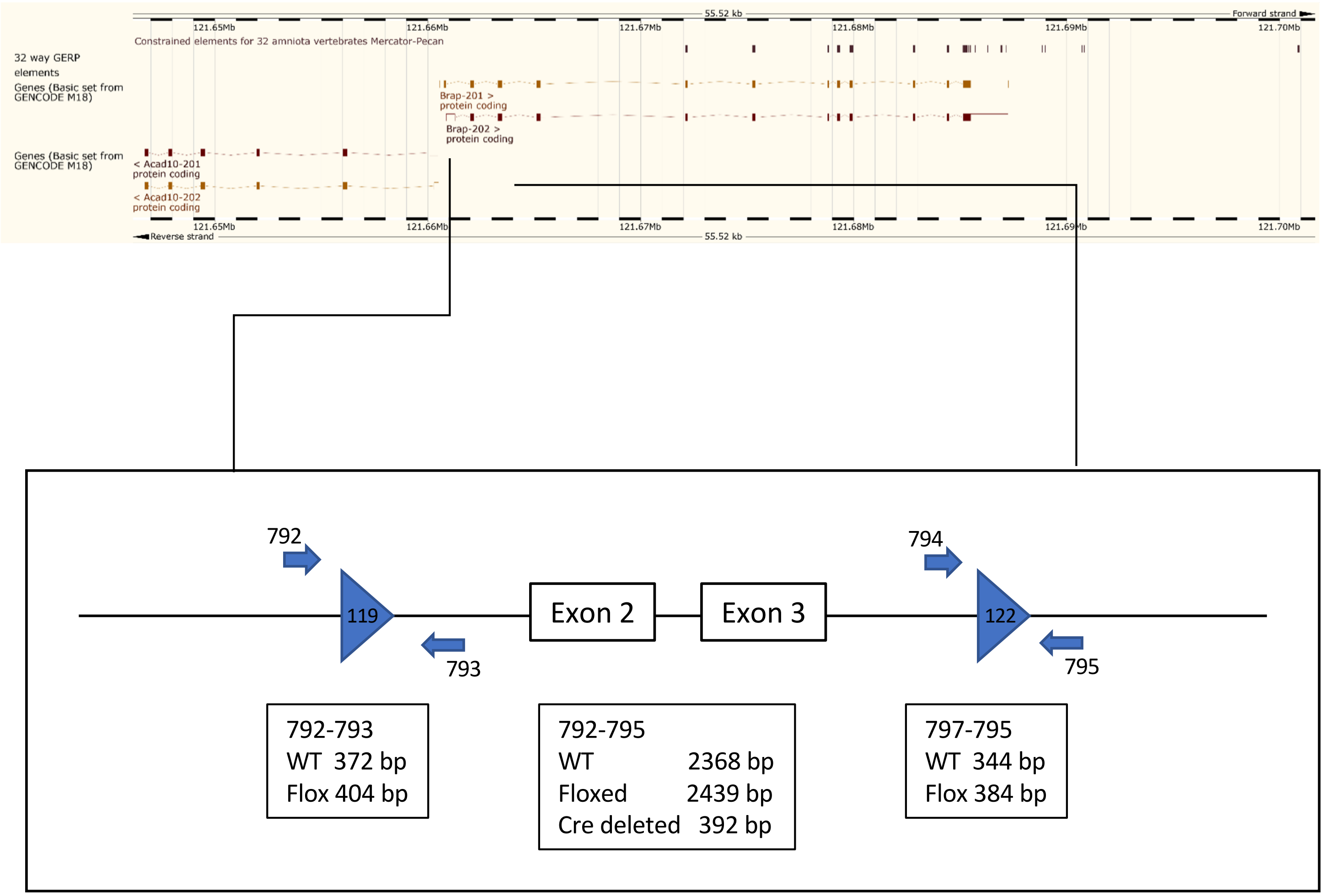

Priest *et al.* Fig S1

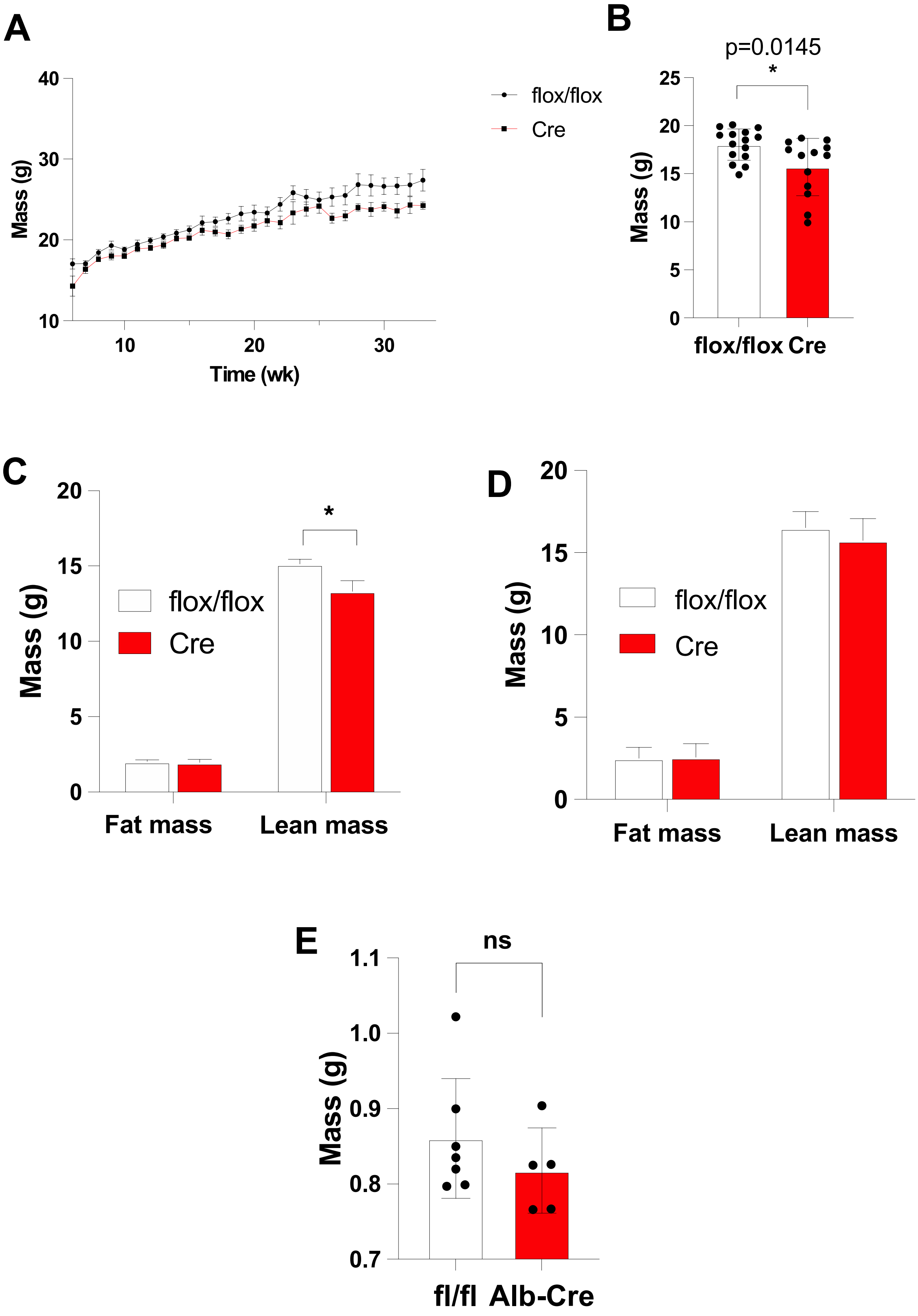

Priest *et al.* Fig S2

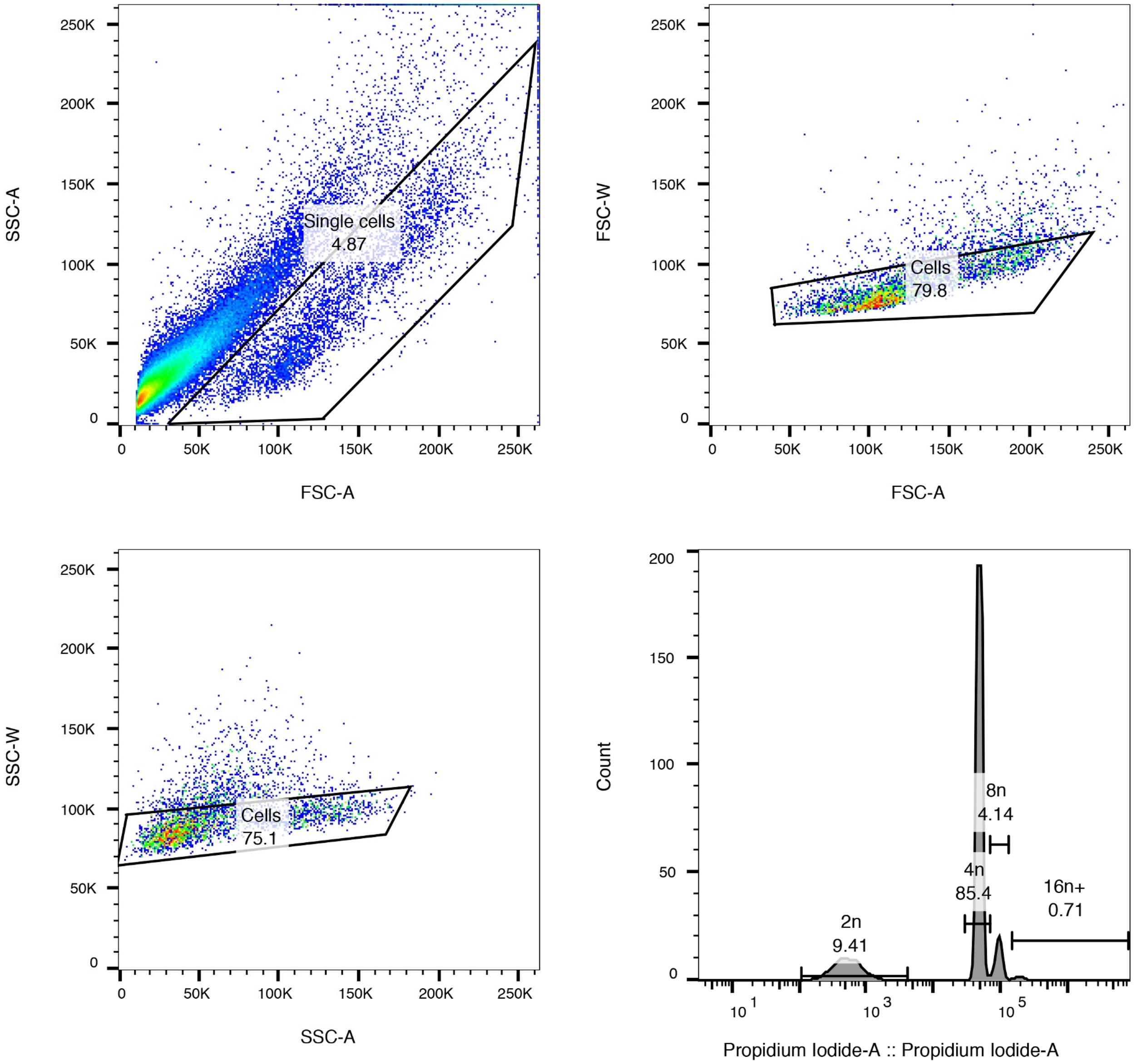

Priest *et al.* Fig S3

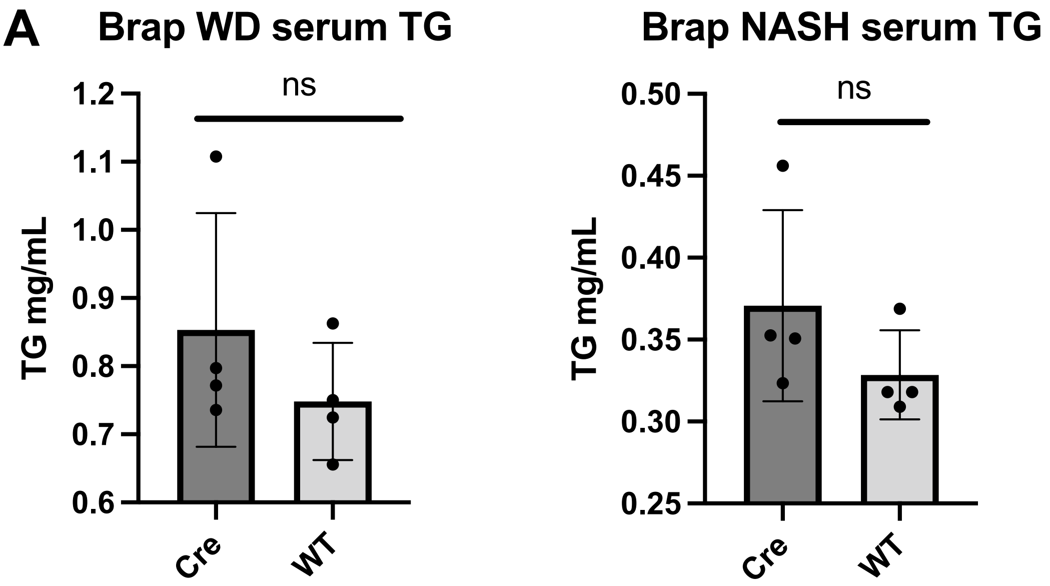

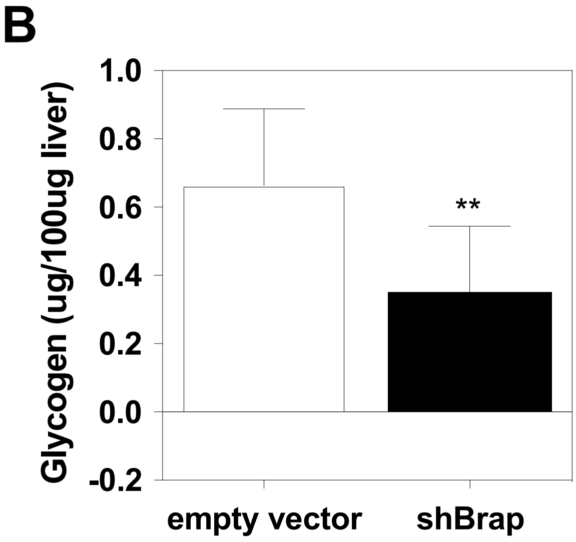

Priest *et al.* Fig S4

REAGENT or RESOURCE	SOURCE	IDENTIFIER
**Antibodies**		
Gapdh	Proteintech	60004-1-Ig
Actin	Sigma	A2066
Cleaved Caspase-3 (D175	Cell Signaling	9661
P-Histone H3 (S10)	Cell Signaling	9701
Surivin (71G4B7)	Cell Signaling	2808
PCNA (PC10)	Cell Signaling	2586
Skp2	Proteintech	15010-1-AP
Mst2	Cell Signaling	3952
Mst1	Cell Signaling	3682
MOB1 (E1N9D)	Cell Signaling	13730
P-MOB1(T35)	Cell Signaling	8699
Yap (D8H1X)	Cell Signaling	14074
P-Yap (S127) (D9W2I)	Cell Signaling	13008
P-Yap (S397) (D1E7Y)	Cell Signaling	13619
P-Yap (S109) (	Cell Signaling	46931
Sav1 (D6M6X)	Cell Signaling	13301
Flag M2	Sigma	
Myc, clone 4A6	EMD Millipore	05-724
HA.11, Clone 16B12	Biolegend	901502

**Experimental diets**		
Chow (13.4%kcal from fat with 209mg/kg cholesterol)	LabDiet	5001
Western Diet (WD: 40%kcal from fat with 2100mg/kg cholesterol)	Research Diets	D12079B
NASH diet	envigo	52 kcal/Fat Diet (C16:0, HVO, AMF, Choline/Met)
**Critical Commercial Assays**		
Infinity Alt (GPT)	Thermo Scientific	TR71121
Infinity AST (GOT)	Thermo Scientific	TR70121
Triglyceride-SL Kit	Sekure Chemistry	236-60
Q5 Site Directed Mutagenesis Kit	New England Biolabs	E0554S
**Experimental Models: Organisms/Strains**		
C57Bl6/J	JAX	664
Albumin Cre	JAX	3574

Oligonucleotides: genotyping primers

Gene	Forward primer sequence	Reverse primer sequence
Cre	GCGGTCTGGCAGTAAAAACTATC	GTGAAACAGCATTGCTGTCACTT
Internal Control	CTAGGCCACAGAATTGAAAGATCT	GTAGGTGGAAATTCTAGCATCATC C
Brap flox	CTGAAAGTCAGTTAAGTTGGCACT	AACAAATTCCTCTCACTTCTTGGC

Oligonucleotides: qPCR primers

Gene	Forward primer sequence	Reverse primer sequence
36B4 (Rplp0)	AGATGCAGCAGATCCGCA	GTTCTTGCCCATCAGCACC
Brap	GCACAGAGAAGTAGAGAACAGAG	GTTGCCACTGAAGAAGGAAATC
F4/80	ACCACAATACCTACATGCACC	AAGCAGGCGAGGAAAAGATAG
Tnfa	CTTCTGTCTACTGAACTTCGGG	CAGGCTTGTCACTCGAATTTTG
Mcp1	TTAAAAACCTGGATCGGAACCAA	GCATTAGCTTCAGATTTACGGGT
CTGF	GGGCCTCTTCTGCGATTTC	ATCCAGGCAAGTGCATTGGTA
Notch2	ATGTGGACGAGTGTCTGTTGC	GGAAGCATAGGCACAGTCATC
Jag1	CCTCGGGTCAGTTTGAGCTG	CCTTGAGGCACACTTTGAAGTA
Ihh	CTCTTGCCTACAAGCAGTTCA	CCGTGTTCTCCTCGTCCTT
Cyr61	GGATCTGTGAAGTGCGTCCT	CTGCATTTCTTGCCCTTTTT
